# Changes in life satisfaction among middle-aged adults living alone over a 12-year span

**DOI:** 10.1371/journal.pone.0295895

**Published:** 2023-12-14

**Authors:** Jaehee Yoon, Jeewuan Kim, Joohyun Chung, Heesook Son

**Affiliations:** 1 Wolchon Elementary School, Seoul, South Korea; 2 Department of Statistics and Data Science, Yonsei University, Seoul, South Korea; 3 College of Nursing, University of Massachusetts Amherst, Amherst, MA, United States of America; 4 Red Cross College of Nursing, Chung-Ang University, Seoul, South Korea; Heidelberg University, GERMANY

## Abstract

This secondary analysis used data collected for the Korean Longitudinal Study of Aging from 2006 to 2018 to examine changes in life satisfaction among middle-aged adults living alone in South Korea. Individuals who were over 45 years of age, lived alone at the time of the first data collection wave, and responded at least twice to the survey over the 12-year study period were included in the final linear mixed model (*N =* 124). Life satisfaction increased for those who had increased assets, were widowed, and had more frequent contact with acquaintances (i.e., once a month and once a week compared with once a year). Life satisfaction decreased as the number of chronic illnesses increased for underweight individuals compared with normal weight or overweight individuals and for depressed versus non-depressed individuals. This study’s findings indicate that increased social support is beneficial for middle-aged marginalized individuals, including those who are economically disadvantaged, have few social interactions, are underweight, and have chronic illnesses.

## Introduction

The rising aging population, decreased marriage rates, and increased divorce rates have caused single-person households to increase worldwide [1, 2]. For instance, the proportion of single-person households in South Korea increased from 15.5% in 2000 to 30.2% in 2019 [3, 4], and some countries are expected to have more than 40% single-person households by 2025–2030 [2]. In particular, middle-aged and older adults comprise 58.7% of all single-person households, with individuals aged 40–49, 50–64, and > 65 years accounting for 19.5%, 27.6%, and 11.6%, respectively [4].

Household composition is associated with numerous social outcomes [2]. In general, living with others is beneficial [5]. Previous studies have demonstrated that living in a single-person household is related to negative outcomes, including low quality of life [6], psychological distress [7], and decreased cognitive function [7]. Additionally, living alone has been found to be related to unhealthy lifestyle behaviors, such as frequent intake of convenience foods [3], smoking [5], physical inactivity [5], and drinking [8].

Life satisfaction is a subjective evaluation of one’s overall life, including psychological and physical well-being [9]. Quality of life is an integrative concept that combines objective human needs with subjective well-being, while life satisfaction can be assessed based on individuals’ responses to questions about life satisfaction variables that they consider important [10]. Life satisfaction is a social indicator for apparent quality of life and has been increasingly used to guide policy decisions in several countries, such as France, the United Kingdom, and the United States [9].

Low life satisfaction is associated with various negative health outcomes, including increased mortality, malnutrition, mood disorders, suicide, and disability, as well as adverse economic outcomes, including income inequality, unemployment, and low socioeconomic status [11–13]. From 2019 to 2021, the average life satisfaction score in South Korea was 5.94 points, which is lower than the OECD average (6.71 points) [14]. Moreover, household composition is related to life satisfaction [15]. Living alone for various reasons (e.g., divorce, death of a spouse, separation from a spouse, and being unmarried) can adversely influence life satisfaction [13, 16].

Middle age is a period of adulthood when life satisfaction is often at its lowest level [16]. As life satisfaction is essential for successful aging [11], middle-aged adults living alone need support to maintain their life satisfaction as they age. Further, health problems decrease the likelihood of middle-aged adults remaining healthy and functional over their remaining lifespan [17]. As middle-aged adults account for 30.5% of those living in single-person households in South Korea [4], examining changes in their life satisfaction and identifying factors associated with these changes are critical tasks. However, most existing research has focused on older adults, and little is known regarding the longitudinal changes in life satisfaction among middle-aged adults living alone.

In general, unlike cross-sectional research, longitudinal studies can detect changes in the characteristics of a target population at both the group and individual levels. A longitudinal study design can potentially detect differences observed in a group that are likely the result of changes or differences over time. Therefore, this study examined changes in life satisfaction over time and factors related to those changes among a sample of middle-aged South Korean adults.

## Materials and methods

### Research design

A longitudinal secondary panel data analysis was used to examine changes in life satisfaction in a sample of middle-aged adults who had lived alone for over 12 years as well as factors associated with these changes.

### Data sources

This study applied biannual data from the Korean Longitudinal Study of Aging (KLoSA) from 2006 to 2018. The KLoSA is a nationally representative longitudinal survey designed to study later life in South Korean individuals aged 45 years or older in households selected by multistage stratified probability sampling based on geographical area [18]. The baseline survey for the KLoSA has been conducted every even-numbered year since 2006. For the KLoSA, trained professional interviewers use a computer-assisted personal interviewing (CAPI) method to directly administer the questionnaires to all participants. As the study was longitudinal, an expected dropout rate was determined to address potential limitations that may negatively affect the generalizability of the findings. The retention rate of the original sample remained stable at 77.6% (*N* = 8596) as of the seventh wave of the survey. In the second wave conducted in 2008, 8688 (84.7%) individuals who participated in the first wave in 2006 were re-interviewed.

**Fig 1:**
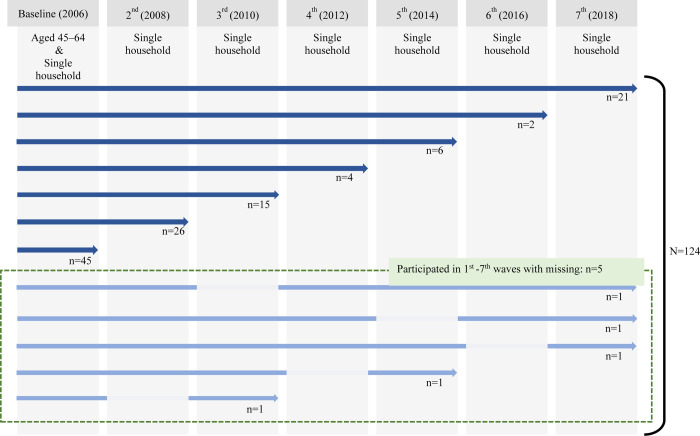
Respondents Included in the analytic sample from the Korean Longitudinal Study of Aging.

The inclusion criteria for the study sample were: (1) being aged between 45 and 64 years when the KLoSA baseline data (*N* = 10 254) were collected in 2006 and (2) living in a single-person household over the study period with observation occurring biannually from 2006 to 2018 (e.g., a respondent completed the first wave survey in 2006, completed the third wave survey in 2010, and so on, and was in a single-person household in both waves). Overall, 124 respondents met the inclusion criteria over the study period (Fig 1). Specifically, 21 respondents completed all surveys from the first to the seventh wave over 12 years, while the remaining 104 individuals did not respond to at least one survey during the study period (Fig 1).

To enhance the quality of the data, the KLoSA uses the CAPI method, as noted above. Using this approach helps reduce errors in responses by organizing the logic and flow of the questionnaire, establishing response ranges, and ensuring consistency in responses [19].

### Study variables

#### Independent variables

Previous research has reported that health, socioeconomic status, and social support are key resources related to life satisfaction [20]. Thus, this study included variables related to those key resources based on responses to the KLoSA survey. Socioeconomic and demographic variables included age, sex, education, marital status, living place, current employment, and assets. Respondents’ net assets were defined as their total assets, including real estate, finances, and businesses, and excluding their total liabilities [18]. Social support was also measured, and included social interaction frequency, such as meeting with acquaintances. Health variables included body mass index (BMI), depressive symptoms, alcohol consumption, smoking status (i.e., non-current vs. current smoker), and number of diagnosed chronic diseases (e.g., hypertension, diabetes, liver disease, and lung disease). BMI was measured using the Quetelet–Kaup Index [21] and categorized as follows: (1) underweight (< 18.5kg/m^2^), (2) normal (18.5–22.9kg/m^2^), (3) overweight (23–25kg/m^2^), and (4) obese (> 25kg/m^2^). Depressive symptoms were assessed using the 10-item Center for Epidemiological Studies Depression Scale (CES-D-10). As a brief screening tool, the CES-D-10 is a 10-item questionnaire that assesses depressive symptoms in the past week [22]. Respondents’ CES-D-10 scores were categorized into two levels: depressed (≥ 3) and not depressed (< 3) [22]. Alcohol consumption was categorized as engaging in high-risk or non-high-risk drinking. High-risk drinking was defined as consuming more than five drinks per occasion for women and more than seven drinks per occasion for men at least twice a week [23]. Based on the heteroscedasticity test results, the regular exercise variable was excluded.

#### Dependent variables

Life satisfaction is a comprehensive concept that encompasses subjective well-being and evaluates individuals’ affective experiences and cognitive assessments of the overall quality of their lives. In this study, it was measured using a single question: “How is your overall life satisfaction?” Responses were rated on a scale ranging from 0 to 100, with higher scores indicating better life satisfaction [18]. The single-item measure of life satisfaction exhibited significant criterion validity compared with the Satisfaction with Life Scale [24].

### Data analysis

Descriptive statistics, including variable distribution and frequency, and repeated measures of mixed models were analyzed using SAS Version 9.4 (SAS Inc., Cary, NC). A survey-weighted analysis was employed to account for the stratification and clustering in the panel dataset. A linear mixed model for longitudinal data was adopted to determine factors associated with life satisfaction over 12 years. Using the Henze–Zirkler test [25], the life satisfaction variable was shown to be normally distributed (Henze–Zirkler T = 0.36, *p* = .37). Specifically, the covariance structure of the random part of the mixed model was used to test the selected fixed and random variables for three reasons: (1) the repeated measurements occurred at fixed intervals, (2) the relationship with time was not of particular interest in our study, and (3) it allowed us to make greater use of incomplete data (i.e., respondents who dropped out or missed scheduled measurements). Consequently, the covariance patterns of the mixed model were used to fit complex covariance patterns that could provide more appropriate fixed effect estimates and standard errors, despite the significance of underweights and their association.

Further, the data collected during the first wave in 2006 were analyzed using Kruskal–Wallis tests, Mann–Whitney U tests, and Spearman correlations to determine factors associated with life satisfaction. Missingness was checked for all study variables, and the missing data rate was less than 3% for all variables except for net assets. The missingness for the variable of net assets was 21% at random (9275 missingness among 43 877 observations). Multiple-imputed datasets from the KLoSA were used to replace the missing values for the net asset variable. The KLoSA conducted multiple imputations based on hot deck to handle missing values in the baseline data. Hot deck imputation involves replacing missing values of one or more variables for a non-respondent (called the recipient) with observed values from a respondent (the donor) who is similar to the non-respondent in relation to characteristics observed in both cases [18, 26].

To enhance the quality of the data and analysis, two biostatisticians on the research team checked the raw data as the first step in data understanding (e.g., checking for potential errors or issues). All analyses and modeling were performed by two biostatisticians. If the two biostatisticians reported different results, the results were re-evaluated.

### Ethical approval

As this study was a secondary data analysis of a national survey, the raw data file was publicly available. Any personal information was de-identified before being provided to the public. The institutional review board where the primary investigator is affiliated approved the analyses (1041078-202106-HRSB-169-01).

## Results

Table 1 illustrates the respondents’ baseline characteristics and the associations with life satisfaction. The respondents’ mean age was 57 years (*SD* = 5.69), and 72% (*n* = 89) were women. Over half of the respondents had less than an elementary school education and lived in a metropolitan area. Approximately 61% (*n* = 76) of the respondents met with acquaintances at least once per week. Over half (53%; *n* = 66) were overweight or obese. Smokers accounted for 28% of the sample and high-risk drinkers for 14%. At baseline, life satisfaction was significantly associated with marital status (χ^2^ *=* 12.3, *p* < .001), age (ρ *=* 0.20, *p* = .03), and current employment status (χ^2^ *=* 18.5, *p* = .05), indicating that life satisfaction was higher for those who were older, widowed, and currently employed than for those who were younger, divorced or never married, and unemployed.

**Table 1:** Baseline characteristics of the respondents and associations with life satisfaction (*N = 124*).

Characteristics	*n* (%) or *M* (*SD*)	χ^2^ or ρ	*p*
**Age (years)**	56.74 (5.69)	0.20	.03
**Sex**			
**Female**	89 (71.77)	8.53	.58
**Male**	35 (28.23)		
**Education**			
**≤ Elementary school**	73 (58.87)	0.53	.77
**Middle or high school**	42 (33.87)		
**≥ College**	9 (7.26)		
**Marital status**			
**Single or never married**	17 (13.71)	12.30	< .001
**Separated or divorced**	36 (29.03)		
**Widowed**	71 (57.26)		
**Living place**			
**Non-metropolitan**	57 (45.97)	15.60	.11
**Metropolitan**	67 (54.03)		
**Currently employed**			
**Yes**	47 (37.9)	18.5	.05
**No**	77 (62.1)		
**Net assets (Won)**	8855.52 (18408.53)	0.11	.28
**Frequency of meeting with acquaintances**			
**Less than once a year**	29 (23.39)	2.70	.26
**Once a week**	76 (61.29)		
**Once a month**	19 (15.32)		
**Body mass index**			
**Underweight (< 18.5kg/m**^**2**^**)**	6 (4.84)	3.33	.34
**Normal (18.5–22.9kg/m**^**2**^**)**	52 (41.94)		
**Overweight (23–25kg/m**^**2**^**)**	32 (25.81)		
**Obese (> 25kg/m**^**2**^**)**	34 (27.42)		
**Number of chronic illnesses**	0.92 (1.06)		
**Smoking status**			
**Non-smoker**	89 (71.77)	12.2	.27
**Smoker**	35 (28.23)		
**Alcohol consumption**			
**Non-high-risk drinking**	110 (88.71)	9.47	.49
**High-risk drinking**	14 (11.29)		
**CES-D-10**			
**Not depressed (< 3)**	75 (60.48)	12.8	.24
**Depressed (≥ 3)**	49 (39.52)		

*Note*. Chi-square tests were used for most of the categorical variables and the Mann–Whitney U test was used for body mass index and 10-item Center for Epidemiological Studies Depression Scale (CES-D-10) scores. Net assets were defined as total assets, including real estate, finances, and businesses, and excluding total liabilities. Smoking status was categorized as non-current versus current smoker. High-risk drinking was considered ≥ five drinks per occasion for women and ≥ seven drinks per occasion for men at least twice per week. Chronic diseases included hypertension, diabetes, liver disease, and lung disease.

**Fig 2:**
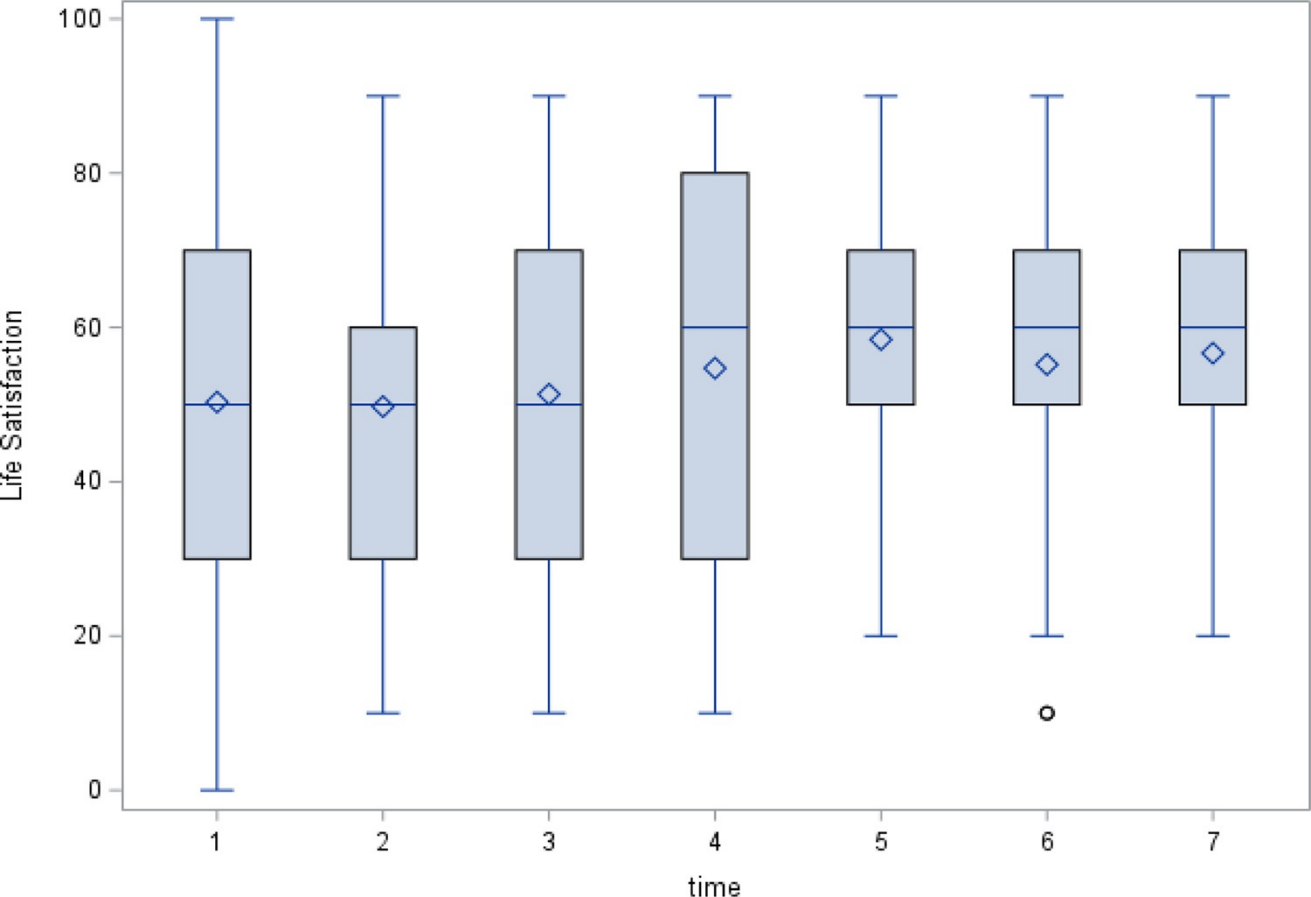
Life satisfaction at each wave.

Life satisfaction increased slightly over time, from 50.3 (*SD* = 26.22) at Wave 1 to 56.7 (*SD =* 26.22) at Wave 7 (Fig 2).

The highest mean life satisfaction score was at Wave 6 (Mean = 61.9, *SD* = 16.3), whereas the lowest mean life satisfaction score was at Wave 1 (Mean = 50.3, *SD* = 26.22). Life satisfaction was more widely dispersed at Wave 1 than at Wave 6 (Fig 2). The percentage of smokers decreased from 28% (*n* = 36 out of 78) at Wave 2 to 8% (*n* = 2 out of 24) at Wave 7. Respondents showed a trend toward depression gradually decreasing over time (40% [*n* = 49] at Wave 1 to 29% [*n* = 7] at Wave 7).

As shown in Table 2, life satisfaction increased over time for those who had more assets (β = 21.50, *p* < .001), were widowed rather than single or divorced (β = 17, *p* < .001), and met more frequently with acquaintances (once a month β = 11.95, *p* < .001, and once a week, *p* < .001, compared with once a year). Life satisfaction decreased over time for individuals who were underweight (β = -9.80, *p* = .04) or obese (β = -5.63, *p* = .05), had a greater number of chronic diseases (β = -2.43, *p* = .03), and had a high depression score (β = -8.21, *p* < .001).

**Table 2:** Changes in life satisfaction and related factors over 12 years.

Characteristics	Unstandardized *B*	SE (*B*)	*t*	*p*
**Age**	0.55	0.20	2.72	<. 001
**Sex**				
**Female**	Ref.			
**Male**	-1.07	3.19	0.33	.74
**Education**				
**≤ Elementary school**	Ref.	.	.	.
**Middle or high school**	3.67	3.00	1.22	.22
**≥ College**	9.60	5.12	1.86	.06
**Marital status**				
**Single or never married**	Ref.	.	.	.
**Separated or divorced**	7.78	4.20	1.85	.07
**Widowed**	17	3.77	4.51	< .001
**Living place**				
**Non-metropolitan**	Ref.	.	.	.
**Metropolitan**	2.96	2.28	1.30	.20
**Currently employed**				
**Yes**	Ref.	.	.	.
**No**	-6.6	2.39	2.76	< .001
**Net assets (won)**	21.50	6.60	3.25	< .001
**Frequency of meeting with acquaintances**				
**Less than once a year**	Ref.			
**Once a week**	11.22	3.77	2.98	< .001
**Once a month**	11.95	3.11	3.84	< .001
**Body mass index**				
**Underweight (< 18.5kg/m**^**2**^**)**	-9.80	4.76	2.06	.04
**Normal (18.5–22.9kg/m**^**2**^**)**	Ref.			
**Overweight (23–25kg/m**^**2**^**)**	2.36	2.84	0.83	.41
**Obese (> 25kg/m**^**2**^**)**	-5.63	2.92	1.93	.05
**Number of chronic illnesses**	-2.43	1.14	2.13	.03
**Smoking status**				
**Non-smoker**	Ref.			
**Smoker**	-2.91	3.71	0.78	.43
**Alcohol consumption**				
**Non-high-risk drinking**	Ref.			
**High-risk drinking**	0.91	5.22	0.18	.86
**CES-D-10**				
**Not depressed (< 3)**	Ref.	.	.	.
**Depressed (≥ 3)**	-8.21	2.45	3.35	< .001
**Intercept**	6.91	12.93	0.53	.59

*Note*. Net assets were defined as total assets, including real estate, finances, and businesses, and excluding their total liabilities. Smoking status was categorized as non-current vs. current smoker. High-risk drinking was considered ≥ five drinks per occasion for women and ≥ seven drinks per occasion for men at least twice a week. Chronic diseases included hypertension, diabetes, liver disease, and lung disease. Abbreviations: CES-D-10, 10-item Center for Epidemiological Studies Depression Scale; Ref., reference group; SE, standard error.

## Discussion

Middle age is the gateway to older age and a critical period for physical and psychosocial health throughout the remainder of one’s life. To the best of our knowledge, this study was the first to examine longitudinal changes in life satisfaction for middle-aged adults over 12 years. We found that life satisfaction for middle-aged adults living alone increased over time, which is consistent with other studies. Life satisfaction has been shown to reach its lowest levels among middle-aged adults before increasing [16], typically displaying a U-shaped pattern across the life span [27]. In this study, the mean life satisfaction score at baseline was 50.32 out of 100, which is a lower score than what has been reported in previous studies [16]. The average life satisfaction score among South Korean older adults has been reported to be 58.5 [28]. Thus, our results are consistent with previous research suggesting that life satisfaction is low in middle-aged adults and increases with age.

Life satisfaction increased for middle-aged adults as their net assets increased. Previous research identified a strong relationship between economic status and life satisfaction, with assets being a significant predictor of life satisfaction in adults aged 55 or older in Singapore [29]. The relationship between assets and subjective well-being (i.e., life satisfaction) can be explained by the need theory, in that assets contribute to fulfilling the most basic of physiological needs, thereby increasing one’s happiness level [30]. Further, assets positively influence various factors, such as financial security, self-esteem, and risk-taking behaviors, and these variables are positively associated with life satisfaction [29]. As economic status was found to be a significant factor related to life satisfaction among middle-aged adults in this study, further attention needs to be paid to economically marginalized individuals in promoting life satisfaction.

Life satisfaction increased over time for widowed individuals compared with those who were single/never married. In a previous 12-year population-based study [16], life satisfaction declined sharply after the death of a spouse but then recovered. Although information on the time since the loss of a spouse was not available in the present study, individuals might have adapted to this loss and recovered life satisfaction over a relatively long period of 12 years.

Life satisfaction increased over time for those who had more frequent interactions with acquaintances, which is consistent with previous research demonstrating that social networks are critical for older adults living alone [11]. One systematic review [31] found that social relationships had an effect on mortality similar to that of smoking cessation interventions, which are recognized as major health promotion strategies.

Further, life satisfaction has been demonstrated to decrease as comorbidities increase in middle-aged adults [32], which is supported by our findings. Previous research indicates that an individual’s number of chronic diseases, general quality of life, and well-being are correlated, suggesting that multiple chronic comorbidities can impair quality of life and psychological well-being [33].

Our study revealed that life satisfaction decreased over time for underweight individuals, which is consistent with previous studies. Considering the limited sample size of underweight individuals (*n* = 6) in this study, a supplementary analysis was conducted to reevaluate the association utilizing BMI as a continuous variable. The results of the supplementary analysis also showed a significant association (β = 1.37, SE = 0.43, t = 3.17, p < 0.01), indicating that these coefficients for the main effects on life satisfaction signify differences between the mean of each level and the overall mean of BMI. Undernutrition has been previously linked to lower life satisfaction [13], regardless of perceived weight [34]. Further, living alone is associated with unhealthy eating habits (i.e., eating alone, skipping meals, increased intake of convenience foods, and decreased fruit and vegetable consumption) and an increased risk of being underweight [35]. The relationship between being underweight and life satisfaction can be explained by the association between being underweight and depression. Being underweight can increase the risk of depression, and depression can negatively affect life satisfaction [36, 37]. In this study, life satisfaction also decreased more over time in the depressed group compared with the non-depressed group.

### Limitations

Our study has several limitations. First, we could not compare changes in life satisfaction between middle-aged adults who lived alone and those who did not because of the limited sample size. Further research should evaluate differences in life satisfaction changes depending on whether middle-aged adults live alone or with others. Further, non-random attrition, whereby the presence in a research sample at a given wave, might directly or indirectly impact the results. Perhaps owing to different health conditions, health concerns, and mortality, health and wealth/economic status were more likely to contribute to the study, which may have important implications for examining changes to life satisfaction over time. Notably, more attrition occurred from Wave 1 (*n* = 124) to Wave 2 (*n* = 78), whereas less attrition occurred from Wave 6 (*n* = 25) to Wave 7 (*n* = 24). Although many factors could result in attrition, such as refusal to participate in the survey or death, a main reason in this study was respondents no longer living alone.

Regarding the findings related to net assets, although multiple imputations were applied, missingness in net assets might have reduced the statistical power. Although the missingness was at random, it might be subject to bias. Further studies should examine the association between net assets and life satisfaction and the related mechanisms among middle-aged adults.

Additionally, the current findings may not provide information on the detailed mechanisms underlying changes in life satisfaction and understanding groups with diverse life experiences. Thus, future studies are required that analyze life satisfaction by subgroup using a sufficient sample size, as well as qualitative studies that explore how living alone influences the life satisfaction of middle-aged adults, how those living alone describe their life satisfaction, and what barriers and challenges they face.

Finally, life satisfaction was measured using a single item. Previous research has reported this to be a reliable and valid instrument [38]; however, given the construct of life satisfaction, a multi-item instrument might be beneficial for comprehensively measuring its aspects. Therefore, further research is necessary to evaluate life satisfaction using a multi-item measure.

## Conclusion and recommendations

The study provides the first evidence on changes over 12 years in life satisfaction and related factors for middle-aged adults. Life satisfaction increased over time for those who had increased net assets, were widowed rather than divorced or single, and frequently met with acquaintances. Individuals’ life satisfaction decreased over time as their number of chronic illnesses increased, and for those who were underweight compared with those who were normal weight or overweight, and were depressed versus non-depressed.

These findings have practical implications for healthcare providers and policymakers regarding ways to improve life satisfaction, indicating the need to provide more attention to vulnerable middle-aged adults. Further research is necessary to identify mechanisms that can explain the changes and related factors in middle-aged adults as observed in this study.

## References

[1] Jacob L, Haro JM, Koyanagi A. Relationship between living alone and common mental disorders in the 1993, 2000 and 2007 National Psychiatric Morbidity Surveys. PLoS One. 2019;14: e0215182. doi: 10.1371/journal.pone.0215182 31042720 PMC6493731

[2] Organization for Economic Co-Operation and Development (OECD). The future of families to 2030: A synthesis report; 2011 [cited 2021 Aug 10]. International Futures Programme. Available from: https://www.oecd.org/futures/49093502.pdf

[3] Statistics Korea. [Single household status in 2020 population and housing census]; 2020 [cited 2021 Aug 10]. Korean. Available from: https://kostat.go.kr/portal/korea/kor_nw/1/1/index.board?bmode=read&aSeq=370806

[4] Statistics Korea. [Single households of statistics 2020]; 2020 [cited 2021 Aug 10]. Korean. Available from: http://kostat.go.kr/portal/korea/kor_nw/1/1/index.board?bmode=read&aSeq=386517

[5] Jeong S, Cho SI. Effects of living alone versus with others and of housemate type on smoking, drinking, dietary habits, and physical activity among elderly people. Epidemiol Health. 2017;39: e2017034. doi: 10.4178/epih.e2017034 29121710 PMC5675988

[6] Chen Y, While AE. Older people living alone in Shanghai: A questionnaire survey of their life experience. Health Soc Care Community. 2019;27: 260–269. doi: 10.1111/hsc.12648 30160058

[7] Mazzuco S, Meggiolaro S, Ongaro F, Toffolutti V. Living arrangement and cognitive decline among older people in Europe. Ageing Soc. 2017;37: 1111–1133. doi: 10.1017/S0144686X16000374

[8] Kim A, Park NL, Lee JA, Park HS. Health behaviors and mental health of Korean young adults from single households: Data analysis from the 5th Korea National Health and Nutrition Examination Survey (2010–2012). Korean J Fam Pract. 2017;7: 667–673. doi: 10.21215/kjfp.2017.7.5.667

[9] Cheung F, Lucas RE. Assessing the validity of single-item life satisfaction measures: Results from three large samples. Qual Life Res. 2014;23: 2809–2818. doi: 10.1007/s11136-014-0726-4 24890827 PMC4221492

[10] Costanza R, Fisher B, Ali S, Beer C, Bond L, Boumans R, et al. An integrative approach to quality of life measurement, research, and policy. Sapiens. 2008;1(1): 17–21. doi: 10.1007/s11136-014-0726-4

[11] Banjare P, Dwivedi R, Pradhan J. Factors associated with the life satisfaction amongst the rural elderly in Odisha, India. Health Qual Life Outcomes. 2015;13: 201. doi: 10.1186/s12955-015-0398-y 26691176 PMC4687085

[12] Muresan GM, Ciumas C, Achim MV. Can money buy happiness? Evidence for European countries. Appl Res Qual Life. 2020;15(4):953–970. doi: 10.1007/s11482-019-09714-3

[13] Ghimire S, Baral BK, Karmacharya I, Callahan K, Mishra SR. Life satisfaction among elderly patients in Nepal: Associations with nutritional and mental well-being. Health Qual Life Outcomes. 2018;16: 118. doi: 10.1186/s12955-018-0947-2 29880002 PMC5992629

[14] Korea Indicator. [Life satisfaction statistics]; 2022 [cited 2023 Jan 4]. Statistics Korea. Korean. Available from: http://kostat.go.kr/portal/korea/kor_nw/1/1/index.board?bmode=read&aSeq=386517

[15] Kooshiar H, Yahaya N, Hamid TA, Abu Samah A, Sedaghat Jou V. Living arrangement and life satisfaction in older Malaysians: The mediating role of social support function. PLoS One. 2012;7: e43125. doi: 10.1371/journal.pone.0043125 22912806 PMC3422218

[16] Qu L, de Vaus D. Life satisfaction across life course transitions; 2015 [cited 2021 Aug 20]. Australian Family Trends (no. 8). Australian Institute of Family Studies. Available from: https://www.researchgate.net/profile/David_De_Vaus/publication/281854745_Life_Satisfaction_across_life_course_transitions/links/55fba2e008aec948c4afafe3/Life-Satisfaction-across-life-course-transitions.pdf

[17] Hughes ME, Waite LJ. Health in household context: Living arrangements and health in late middle age. J Health Soc Behav. 2002;43: 1–21. doi: 10.2307/3090242 11949193 PMC1440422

[18] Korea Employment Information Service [KEIS]. [*KloSA user’s guide*]; 2020 [cited 2021 Aug 20]. Employment Survey Analysis System. Korean. Available from: https://survey.keis.or.kr/klosa/klosaguide

[19] Shin HG, Lee HJ. CAPI and higher data quality: The case of KLoSA and Blaise CAPI Programme. Surv. Rev. 2006;7(2): 71–95. Available from: https://koreascience.kr/article/JAKO200634514812490

[20] Bishop A, Martin P, Poon L. Happiness and congruence in older adulthood: A structural model of life satisfaction. Aging Ment Health. 2006;10(5): 445–453. doi: 10.1080/13607860600638388 16938680

[21] Korean Health Promotion and Development Institute. [2*021 Community Health Promotion Project Guide*]; 2021 [cited 2021 Aug 20]. Health and Welfare Administration. Korean. Available from: https://www.khealth.or.kr/kps/publish/view?menuId=MENU00890&page_no=B2017003&pageNum=1&siteId=&srch_text=&srch_cate=&srch_type=&str_clft_cd_list=&str_clft_cd_type_list=&board_idx=10685

[22] Andresen EM, Malmgren JA, Carter WB, Patrick DL. Screening for depression in well older adults: Evaluation of a short form of the CES-D. Am J Prev Med. 1994;10: 77–84. doi: 10.1016/S0749-3797(18)30622-6 8037935

[23] Ministry of Health and Welfare, Fund Policy Team. [4th National Health Promotion Plan, 2019 Trend Report]; 2019 [cited 2021 Aug 23]. Health and Welfare Administration. Korean. Available from: https://www.khealth.or.kr/board/view?pageNum=1&rowCnt=8&no1=31&linkId=1000883&menuId=MENU00829&schType=0&schText=&boardStyle=Gallery&categoryId=&continent=&country=&contents1=

[24] Diener E, Chan MY. Happy people live longer: Subjective well-being contributes to health and longevity. Appl. Psychol. 2011;3(1): 1–43. doi: 10.1111/j.1758-0854.2010.01045.x

[25] Henze N, Zirkler B. A class of invariant consistent tests for multivariate normality. Commun Stat Theory Methods. 1990;19: 3595–3617. doi: 10.1080/03610929008830400

[26] Andridge RR, Little RJ. A review of hot deck imputation for survey non-response. Int Stat Rev. 2010;78: 40–64. doi: 10.1111/j.1751-5823.2010.00103.x 21743766 PMC3130338

[27] Blanchflower DG, Oswald AJ. Is well-being U-shaped over the life cycle? Soc Sci Med. 2008;66(8): 1733–1749. doi: 10.1016/j.socscimed.2008.01.030 18316146

[28] Lee SE. Factors associated with life satisfaction among older adults in Korea according to living arrangements. Korean J Adult Nurs. 2016;28: 659–668. doi: 10.7475/kjan.2016.28.6.659

[29] Hong SI, Han CK. Asset impacts on life satisfaction in an asset-rich country: Focusing on older adults in Singapore. Soc Indic Res. 2014;118: 125–140. doi: 10.1007/s11205-013-0410-z

[30] Howell RT, Howell CJ. The relation of economic status to subjective well-being in developing countries: A meta-analysis. Psychol Bull. 2008;134: 536–560. doi: 10.1037/0033-2909.134.4.536 18605819

[31] Holt-Lunstad J, Smith TB, Layton JB. Social relationships and mortality risk: A meta-analytic review. PLoS Med. 2010;7: e1000316. doi: 10.1371/journal.pmed.1000316 20668659 PMC2910600

[32] Friedman EM, Ryff CD. Living well with medical comorbidities: A biopsychosocial perspective. J Gerontol B Psychol Sci Soc Sci. 2012;67: 535–544. doi: 10.1093/geronb/gbr152 22377799 PMC3441187

[33] Wikman A, Wardle J, Steptoe A. Quality of life and affective well-being in middle-aged and older people with chronic medical illnesses: A cross-sectional population based study. PLoS One. 2011;6: e18952. doi: 10.1371/journal.pone.0018952 21559485 PMC3084723

[34] Herman KM, Hopman WM, Rosenberg MW. Self-rated health and life satisfaction among Canadian adults: Associations of perceived weight status versus BMI. Qual Life Res. 2013;22: 2693–2705. doi: 10.1007/s11136-013-0394-9 23539466

[35] Tani Y, Kondo N, Takagi D, et al. Combined effects of eating alone and living alone on unhealthy dietary behaviors, obesity and underweight in older Japanese adults: Results of the JAGES. Appetite. 2015;95: 1–8. doi: 10.1016/j.appet.2015.06.005 26116391

[36] Jung SJ, Woo HT, Cho S, Park K, Jeong S, Lee YJ, et al. Association between body size, weight change and depression: Systematic review and meta-analysis. Br J Psychiatry. 2017;211: 14–21. doi: 10.1192/bjp.bp.116.186726 28428339

[37] Gigantesco A, Fagnani C, Toccaceli V, Stazi MA, Lucidi F, Violani C, et al. The relationship between satisfaction with life and depression symptoms by gender. Front Psychiatry. 2019;10: 419. doi: 10.3389/fpsyt.2019.00419 31258495 PMC6588028

[38] An HY, Chen W, Wang CW, Yang HF, Huang WT, Fan SY. The relationships between physical activity and life satisfaction and happiness among young, middle-aged, and older adults. Int J Environ Res Public Health. 2020;17: 4817. doi: 10.3390/ijerph17134817 32635457 PMC7369812

